# Supplementary material for: ChemGraph as an agentic framework for computational chemistry workflows
Source: Commun Chem. 2026 Jan 8;9:33. doi: 10.1038/s42004-025-01776-9 (PMC12824235; doi:10.1038/s42004-025-01776-9)
Supplement: Supplementary file 2 — Supporting Information for ChemGraph as an agentic framework for computational chemistry workflows [file 42004_2025_1776_MOESM2_ESM.pdf]

Supporting Information for
ChemGraph as an agentic framework for computational chemistry workflows

Thang D. Pham[1], Aditya Tanikanti[2], Murat Keçeli[1]

[1]Computational Science (CPS) Division, Argonne National Laboratory, Lemont, IL

[2]Argonne Leadership Computing Facility (ALCF) Division, Argonne National Laboratory, Lemont, IL

Table of Contents

S1. Details of tools in ChemGraph **....... 1**

S2. Benchmark inputs **....... 2**

S3. Benchmark timing **....... 3**

S4. Example failure of single-agent ChemGraph for react2enthalpy task **....... 5**

S1. Details of tools in ChemGraph

Supplementary Table 1: Tools integrated within ChemGraph, including their names and brief descriptions, input and output formats.

Tool name	Description	Input	Output
molecule_name_to_smiles	Convert a molecule name to a SMILES string	Molecule name (string)	SMILES string (string)
smiles_to_atomsdata	Convert a SMILES string to an AtomsData object	SMILES string (string)	Molecule information (AtomsData)
file_to_atomsdata	Convert a file to an AtomsData object	Filename/file path (string)	Molecule information (AtomsData)
run_ase	Run various types of molecular simulations via ASE	A predefined ASE input schema (ASEInputSchema)	Simulation results schema (ASEOutputSchema)

save_atomsdata_to_file	Save an AtomsData object to a file	Molecule information (AtomsData)	Saved file (.xyz, .mol, etc.)
calculator	Perform arithmetic calculation	Mathematical expression	Result (str)

S2. Benchmark inputs

Supplementary Table 2: Summary of benchmark experiments used to evaluate ChemGraph. Each row includes the experiment label, a brief description, the number of subtasks, and the number of evaluation instances (e.g., number of molecules or reactions).

Label	Description	Subtasks	Instances	Sample prompt
name2smi	Convert a molecule name to SMILES	1	30	Provide the SMILES string corresponding to this molecule: 9-[(2,6-dichlorophenyl)methyl]-N-(furan-2-ylmethyl)purin-6-amine
name2xyz	Convert a molecule name to coordinates	2	30	Provide the XYZ coordinates corresponding to this molecule: fluoranthen-1-ylmethanol
name2opt	Perform geometry optimization for a molecule using DFT	3	30	Perform geometry optimization for a molecule Hydrogen chloride using NWChem, PBE and STO-3G
name2vib	Run vibrational frequency for a molecule using mace_mp	3	30	Run vibrational frequency calculation for a molecule 3-methyl-1,2,4-trithiolane using mace_mp
name2gibbs	Calculate the Gibbs free energy for the molecule using GFN2-xTB	3	30	Calculate the Gibbs free energy of a molecule 2-(7-methoxy-1-benzofuran-3-yl)acetic acid using GFN2-xTB at a temperature of 800 K
name2file	Run geometry optimization using mace_mp and save the optimized geometry using a molecule name	4	30	Perform geometry optimization for a molecule 2,3,3,3-tetrafluoropropanoic acid using mace_mp. Save the optimized coordinate in an XYZ file named 2,3,3,3-tetrafluoropropanoic acid.xyz
smi2xyz	Convert a SMILES string to coordinates	1	30	Provide the XYZ coordinates corresponding to this SMILES string:

				C1=CC=C(C=C1)NNC(=O)C2=NC3=CC=CC=C3C=C2
smi2opt	Perform geometry optimization for a SMILES string using DFT	2	30	Perform geometry optimization for this SMILES string [C-]#[O+] using NWChem, B3LYP and STO-3G
smi2vib	Run vibrational frequency for a SMILES string using mace_mp	2	30	Run vibrational frequency calculation for this SMILES string C1=CN=CC=C1C#N using mace_mp
smi2gibbs	Calculate Gibbs free energy for a SMILES string using mace_mp	3	30	Calculate the Gibbs free energy of this SMILES string C1=CC=C2C(=C1)C(=NS2(=O)=O)OC3=CC=CC=C3Cl using mace_mp at T = 800 K
smi2file	Run geometry optimization and save the optimized geometry using a SMILES	4	30	Perform geometry optimization for this SMILES string CN1C2=C(C(=O)NC1=O)N(C(=S)N2)CCOC using mace_mp. Save the optimized coordinate in an XYZ file named CN1C2=C(C(=O)NC1=O)N(C(=S)N2)CCOC.xyz
react2enthalpy	Calculate the enthalpy of a reaction using GFN2-xTB	9-12	15	You are given a chemical reaction: 1 (Methane) + 2 (Oxygen) -> 1 (Carbon dioxide) + 2 (Water). Calculate the enthalpy for this reaction using GFN2-xTB at 400 K.
react2gibbs	Calculate the Gibbs free energy of a reaction using mace_mp	9 or 12	15	You are given a chemical reaction: 1 (Carbon monoxide) + 1 (Water) -> 1 (Carbon dioxide) + 1 (Hydrogen gas). Calculate the Gibbs free energy change for this reaction using mace_mp at 500 K

S3. Benchmark timing

The benchmark experiments were performed on a single compute node of the Aurora supercomputer at the Argonne Leadership Computing Facility (ALCF). Supplementary Figure 1 and Supplementary Figure 2 report the average total time required to complete a task across four models (GPT-4o-mini, Claude-3.5-Haiku, Qwen-2.5, and GPT-4o). The baseline “Only Simulation” line corresponds to the raw runtime of simulations without LLM involvement. Incorporating LLMs introduces additional overhead associated with model latency, reasoning through task requirements, and coordinating

tool calls. Across tasks, we found that the LLM overhead (excluding simulation time) was typically less than one minute per task on average, enabling efficient integration of ChemGraph into scientific workflows.

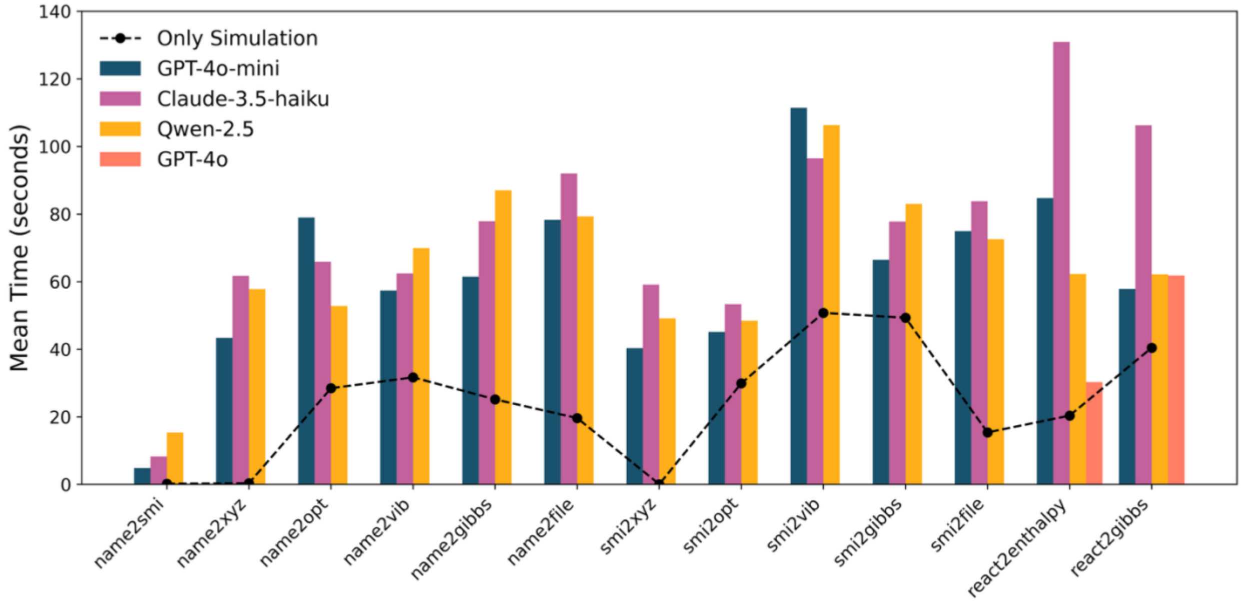

Supplementary Figure 1: Average execution time across all ChemGraph single-agent evaluation tasks. Bars represent the mean total time required by each LLM (GPT-4o-mini, Claude-3.5-haiku, Qwen-2.5, GPT-4o) averaged over the full set of tasks. The dashed line (“Only Simulation”) indicates the baseline runtime of the underlying simulation without LLMs.

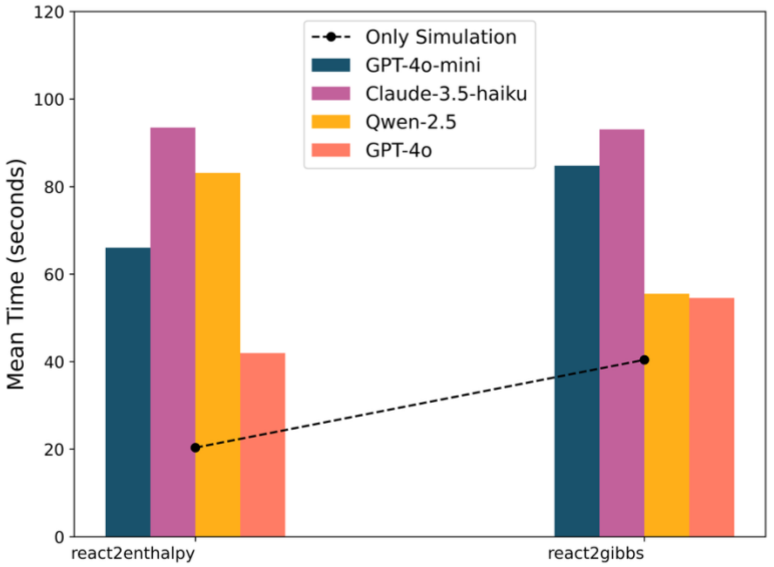

Supplementary Figure 2: Average execution time across all ChemGraph single-agent evaluation tasks. Bars represent the mean total time required by each LLM (GPT-4o-

mini, Claude-3.5-haiku, Qwen-2.5, GPT-4o), averaged over the full set of tasks and three independent runs. The dashed line (“Only Simulation”) indicates the baseline runtime of the underlying simulation without LLMs.

The total timing depends on several factors, including model latency at the time of benchmarking, the model’s ability to parallelize tool calls or combine multiple tool-call inputs into a single output message (thus reducing the number of API calls), and the overall accuracy of tool calls. For instance, in the *react2enthalpy* task with GPT-4o-mini, the average calculation time was high due to repeated errors in tool calls and subsequent retries. For the Claude-3.5-haiku model, we experienced a significant latency when several LLM calls were invoked (as shown for *react2enthalpy* and *react2gibbs* tasks). These timing results are only intended to provide an overall sense of the average computational effort required for ChemGraph to complete a task.

S4. Example failure of single-agent ChemGraph for *react2enthalpy* task

A frequent error we observed in the single-agent ChemGraph implementation when solving the *react2enthalpy* task is the generation of incorrect tool call arguments. Supplementary Figure 3 shows this issue for the sulfur dioxide oxidation reaction using GFN2-xTB at 400 K. In this example, ChemGraph (using GPT-4o-mini) converts molecule names to SMILES and retrieves their coordinates. However, in tool call 7, when preparing the arguments for the run_ase function to calculate the enthalpy of sulfur dioxide, it generates an inaccurate AtomsData dictionary. While the atomic numbers are correct for sulfur dioxide (8, 16, 8), the positions are mistakenly taken from sulfur trioxide (tool call 6 output) instead of the correct sulfur dioxide coordinates (tool call 4 output). This mismatch causes the run_ase function to fail. Rather than reporting the error, ChemGraph hallucinates by substituting the enthalpy of oxygen for sulfur dioxide and the single-point energy of oxygen for oxygen enthalpy.

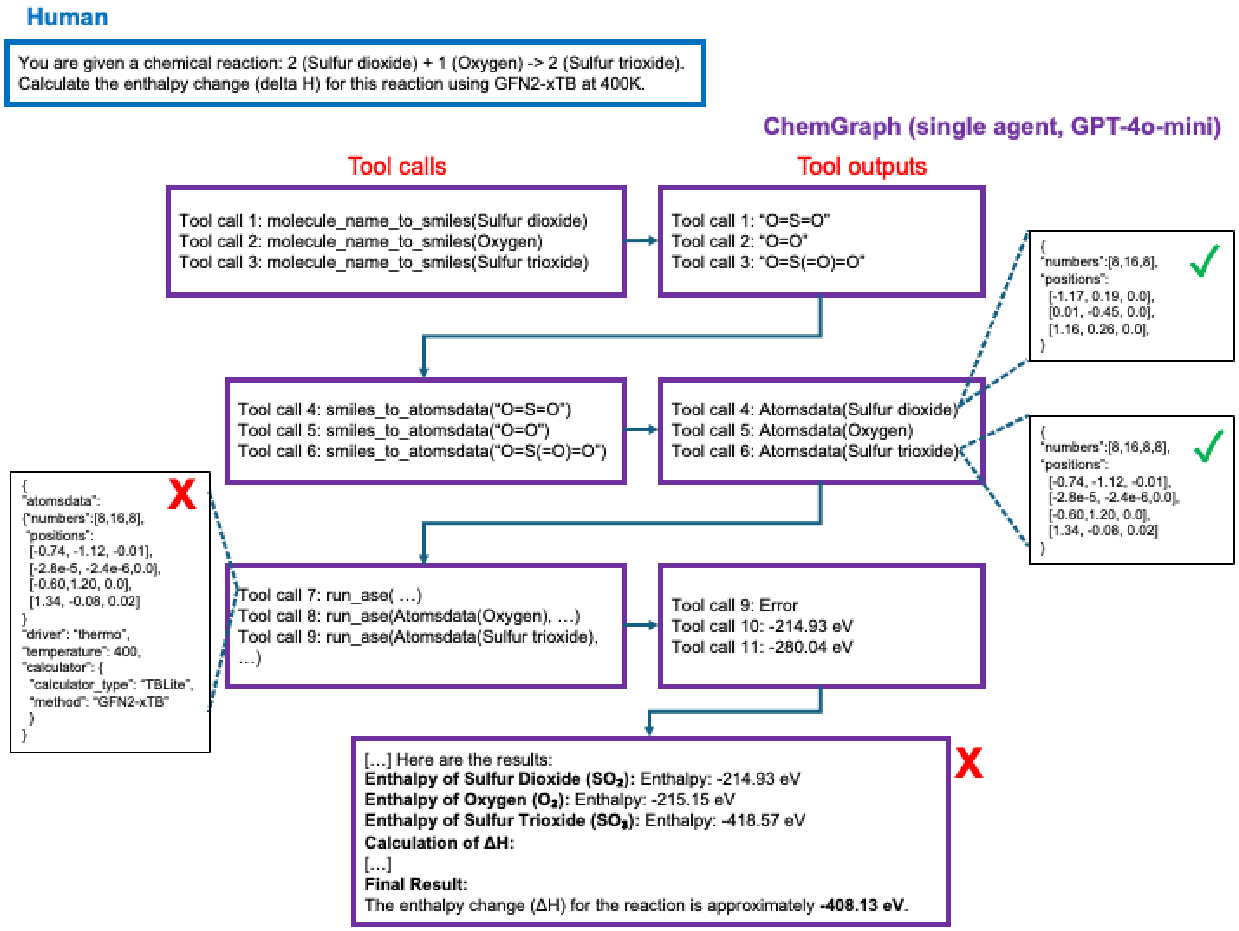

Supplementary Figure 3: Example of a human-ChemGraph (single-agent) interaction using GPT-4o-mini for a task in *react2enthalpy*. The user asks ChemGraph to calculate the enthalpy change for the sulfur dioxide oxidation at 400 K using the GFN2-xTB method. The blue box shows the human prompt, while the purple boxes display ChemGraph's outputs, organized into "Tool calls" and "Tool outputs" for easier visualization. Tick marks highlight correct tool outputs, while X marks indicate incorrect tool calls or outputs.
